# Managing pests by increasing predators through late termination of cover crops

**DOI:** 10.1002/ps.70898

**Published:** 2026-05-20

**Authors:** Jared S Adam, John M Wallace, John F Tooker

**Affiliations:** ^1^ Department of Entomology The Pennsylvania State University University Park PA USA; ^2^ Department of Land Resources and Environmental Sciences Montana State University Bozeman MT USA; ^3^ Department of Plant Science The Pennsylvania State University University Park PA USA

**Keywords:** biological control, cereal rye, natural enemies, pest control

## Abstract

**BACKGROUND:**

Adding cover crops to crop rotations can improve the sustainability and ecosystem functioning of agroecosystems. Establishing cash crops into a living cover crop, or planting green, in agronomic systems can provide another set of functional benefits, including improved control of weeds and invertebrate pests, potentially reducing the need for preventative pest management strategies. Here, we studied the role of delayed cover‐crop termination in managing invertebrate pests through an increase in natural enemies in corn and soybeans. Our work is among the first studies focused on timing of termination of a cover crop and the role that timing has in managing invertebrate pests.

**RESULTS:**

In both crops, we found that delaying termination reduces pest incidence and damage without affecting yield. Although crop type influenced predator and pest responses, neither crop experienced an increase in pest pressure or damage with later timing of termination. Regardless of treatment, soybean harbored greater numbers of natural enemies than corn, a result likely influenced by abiotic conditions associated with crop morphology.

**CONCLUSION:**

Our study shows that by delaying cover‐crop termination, growers can effectively increase natural‐enemy populations and reduce the need for chemical controls. © 2026 The Author(s). *Pest Management Science* published by John Wiley & Sons Ltd on behalf of Society of Chemical Industry.

## Graphical abstract

Adding cover crops to crop rotations can improve the sustainability and ecosystem functioning of agroecosystems. By delaying cover‐crop termination, growers can effectively increase natural enemy populations and reduce the need for chemical controls.

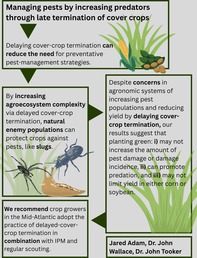

## 1 INTRODUCTION

Due to environmental concerns about runoff of sediment and nutrients from crop fields in many watersheds, federal and state agencies (e.g., USDA‐NRCS, Conservation Districts) and other institutions (e.g., extension programs at land‐grant universities) have made concerted efforts to protect watersheds by encouraging field‐crop farmers to reduce tillage and adopt cover crops. This effort has been particularly strong and successful in the Mid‐Atlantic states of the U.S., where efforts have made this region a national leader in adoption of conservation‐tillage and no‐till farming in agronomic systems. No‐till and cover‐cropped systems reduce erosion, limiting entry of soil and associated nutrients into local streams and reducing amounts of pollutants that are carried to the Chesapeake Bay. In addition to reducing erosion, no‐till farming helps build soil structure, maintains or increases soil organic matter, and increases water‐use efficiency and storage of water from precipitation.^1^ Eliminating tillage can also help build abundance of soil‐associated natural enemies that can aid in control weeds and invertebrate pests, some of which can be fostered by no‐till systems.^2^, ^3^, ^4^, ^5^, ^6^, ^7^, ^8^

Cover crops can amplify many of the benefits of no‐till farming.^9^ Moreover, the combination of cover crops and no‐till can improve agroecosystem resiliency and environmental quality; potential improvements include further increasing organic matter, mitigating and adapting agroecosystems to changes to the climate, such as drought, and further improving habitats for natural enemies.^1^, ^10^, ^11^, ^12^ A potential agronomic tradeoff of cover crops is that they can create cooler and wetter habitats that slow growth or foster pest populations.^13^, ^14^ Specifically, cool, wet conditions created by cover crops can increase populations of some invertebrate pest species.^3^, ^15^ However, some of these concerns can be moderated by maintaining healthy communities of natural enemies.^16^

Traditionally, cover crops have been terminated 7–21 days prior to cash‐crop planting to conserve soil moisture and nutrients, break pest life cycles, and improve planting conditions. Delaying termination of cover crops to shortly after cash‐crop planting is an emerging practice known as ‘planting green’ because the cash crop is established into a living, green cover crop. Allowing cover crops to grow later into spring provide some benefits, including increasing cover‐crop biomass, which can increase water retention, reduce soil erosion,^9^, ^17^ improve soil quality,^9^ reduce nutrient losses,^18^, ^19^ and decrease weed^20^ and invertebrate pest pressures by increasing abundance of natural enemies.^4^ The potential of cover crops to help increase natural‐enemy populations is of particular interest for managing slug (Mollusca: Gastropoda: Agriolimacidae, Arionidae) populations in no‐till systems due to their potential to damage crops in spring and fall in no‐till production.^3^, ^21^, ^22^

Planting green, however, can be accompanied by several concerns. Producer surveys suggest that barriers to adoption of planting green include a lack of measurable economic gain, increased labor costs, allelopathic effects from some cover crop species, yield reduction from competition of cover crops with cash crops, or that cover crops can create field conditions that are too dry or wet.^23^ In addition to production concerns, the moist, shaded habitat and alternative food source provided by cover crops hold potential to increase abundance of invertebrate pests, particularly slugs, in regions that receive enough precipitation to allow them to be abundant.^3^ Lastly, the timing and logistics of planting green can be complicated and growers can experience yield reductions if they do not have their planter set up properly or due to combinations of factors such as delayed germination from poor soil‐seed contact and increase in pest abundance.^24^

To benefit from the pest‐management potential of a conservation‐based system, growers would need to adopt integrated pest management (IPM) to limit insecticide use to only economically justified applications to allow natural‐enemy communities to grow. This combination of no‐till, cover crops, and IPM can lead to a diverse natural‐enemy system in which crop plants are protected by invertebrates, like carabid beetles, in some cases more effectively than control provided by insecticides.^25^, ^26^ By embracing high diversity‐IPM systems, growers can reduce herbivore damage to their crops, increase natural‐enemy communities, and maintain, if not improve, economic and environmental sustainability.^25^, ^27^

With growing popularity of delaying cover‐crop termination and planting green, there is a need to understand effects of delayed cover‐crop termination on pest control in no‐till agronomic systems. In this paper, we quantify effects of differential timing of termination of a cereal‐rye (*Secale cereale* L.) cover crop on pest and predator communities in a no‐till corn and soybean system in Pennsylvania. To do this, we imposed three cover‐crop treatments and a no‐cover‐crop treatment prior to corn and soybean. Our treatments varied in termination timing, ranging from 28 days before cash‐crop planting to several days after cash‐crop planting. We hypothesized that by increasing cover‐crop biomass (i.e., ecosystem complexity) we would see in the late‐terminated, high‐biomass treatment: (i) higher natural‐enemy populations and more prey killed, (ii) lower damage from invertebrate pests, and (iii) competitive crop yields among treatments, especially when cover‐crop biomass was higher.

## 2 MATERIALS AND METHODS

### 2.1 Study location and experimental design

We conducted a three‐year field experiment (2021–2023) at Penn State's Russell E. Larson Agricultural Research Center (Pennsylvania Furnace, Pennsylvania, U.S.A.; 40.720064, −77.934317). In a long‐term no‐till field (> 10 years), we established a single‐factor experiment, arranged in a randomized complete block design with four cover‐crop treatments and five replicates. Plots were 12 × 15 m. Cover‐crop treatments varied by the timing of termination of a cereal‐rye cover crop and included (i) a no‐cover‐crop control (referred to in figures as No CC and in text as no‐cover‐crop) and cover crop terminations of: (ii) 14–28 days pre‐planting (DPP; referred to here as ‘early terminated’), 3) 9–14 DPP (‘late terminated’), and 4) 0–3 days after planting (DAP; ‘planting green’) of the cash crop. For the no‐cover‐crop treatment, field residue consisted of any leftover debris from the previous crop; we made no efforts to remove lingering residue prior to cover‐crop or cash‐crop establishment. Our goal for the late‐terminated treatment was 3–7 DPP, but due to field conditions each year we had to terminate for this treatment between 9 and 14 DPP. Cover crops were terminated using glyphosate (1.27 kg ai ha^−1^) and ammonium sulfate (2.5% v/v) using water as a carrier at 140 L ha^−1^. Based on abiotic conditions, planting dates of cover and cash crops, and termination dates varied annually (Supporting Information, Table S1; For soil type information, see Supporting Information, Table S1(a)). We established a 3‐m border of cover crop around the experiment and 9‐m alleys between blocks. Borders and alleys followed the planting‐green schedule for termination and cash‐crop planting (Supporting Information, Fig. S1). We used alley widths of 9 m to facilitate movement of machinery near plots. There were no alleys between plots, but plot dimensions were larger than typical to reduce the risks of spillovers from arthropods spilling into adjacent plots.

In one field in 2021, we established corn in the plots that had received the four cover‐crop treatments (cover crops were established in fall 2020). In 2022, we established cash crops in two fields: the first followed the corn from 2021 with soybean using the same plots and treatments (cover‐crop treatments were established in fall 2021). In a second field, we established corn with 20 plots in the same design described above (this second field was also long‐term no‐till [>10 years]; cover‐crop treatments were established in fall 2021). In 2023, again we studied two fields: we planted soybeans in the second field from 2022 (previously corn; cover‐crop treatments established in fall 2022) and planted corn in a third long‐term no‐till field (same experimental design and plot dimensions as the other two fields) where we had established the cover‐crop treatments in fall of 2022. For all fields, we planted cash crops with 76.2 cm spacing between rows and used annual rotations of corn followed by soybean or soybean followed by corn. All corn seeds were treated with fungicidal seed treatment (Maxim Quattro; active ingredients: Fludioxonil, mefenoxam, azoxystrobin, thiabendazole) but no insecticidal seed treatments; we also avoided other insecticides. We managed insect and slug pests with integrated pest management (IPM), but pest populations in the plots did not exceed thresholds in any year; therefore, we did not apply insecticide or molluscicide to any plots.

For fields one and two, we practiced a corn‐soybean sequence, where soybean followed corn from the previous year, simulating a rotation commonly used by Pennsylvania growers. Adding soybeans after corn gave us the opportunity to document longer‐term effects of planting green on pest and natural‐enemy populations and communities. After year 3, field one had experienced a cash crop rotation of corn‐soybean (2021–2022), field two experienced corn‐soybean (2022–2023), whereas field three only experienced corn (2023). For all fields, we applied the same cover‐crop treatment to plots with the same experimental layout as the previous year. For the legacy pairs of 2021 corn – 2022 soybean and 2022 corn – 2023 soybean, we collected the following data: cover crop biomass, yield, slug counts, and attacks on sentinel prey. Additionally, in 2022 corn – 2023 soybean we also conducted pitfall sampling. We did not collect pitfall samples in corn in 2021.

To characterize effects of termination date on cover‐crop productivity, we harvested aboveground biomass from each plot 1–2 days prior to termination. From four randomly positioned quarter‐meter quadrats per plot, we harvested cover‐crop biomass 2.5 cm above the soil surface, dried it in an oven (70 °C) for 5–7 days, and then weighed it.

### 2.2 Damage from pests and pest abundance

To understand the influence of insect and slug populations on corn establishment and growth, we evaluated plant populations and early season herbivory in all 3 years of the experiment (2021–2023). Because of time constraints and minimal insect feeding, we did not collect similar data in soybean. When corn plants reached growth stage V3 and V5, we established 3‐m long transects in three randomly selected rows within each plot. For each plant along this transect, we characterized the damage caused by invertebrates and the type of damage, including slugs (Mollusca: Stylommatophora: Agriolimacidae, Arionidae), true armyworm (Insecta: Lepidoptera: Noctuidae: *Mythimna unipuncta*), black cutworm (Noctuidae: *Agriotis ipsilon*), brown marmorated stink bug (Hemiptera: Pentatomidae: *Halyomorpha halys*), damage from multiple pests, or ‘other herbivores’ for each plant. Because slugs feed using a scraping motion, slug damage is distinct from the chewing damage of caterpillars or that of other types of pests. Their damage looks like shredding damage that runs parallel to corn leaf veins. Slug damage is often accompanied by a slime trail that aids in identification. True armyworm damage usually starts from the leaf margin and works inward, looks ragged and rarely crosses the mid‐vein. Young black cutworms bore through the rolled leaves of young plants, so their damage looks like a line of holes; older caterpillars cut plants off at the soil level. For brown marmorated stink bug, a piercing‐sucking herbivore, we identify its damage by unique scaring left behind by its mouthparts.^28^ We quantified damage as the proportion of plants damaged out of the total assessed. Within these same transects, we also assessed incidence of damage, which is represented as the proportion of damaged plants per damage type (e.g., proportion of plants with slug damage). Lastly, for all pests, we rated damage using a scale from 0–4: 0 (no damage), 1 (<25% defoliation/ scarring (for brown marmorated stink bug)), 2 (25–50% defoliation/ scarring), 3 (50–75% defoliation/ scarring), 4 (>75% defoliation/ scarring).^29^

In addition to assessing damage they caused, we also quantified slug populations weekly using shelter traps made from pieces (30 × 30 cm) of white roofing shingles (cut from Owens Corning Rolled Roofing Material; color: Shasta White). We quantified slug populations in corn plots in 2021, and corn and soybean plots in 2022 and 2023. Between crop rows 6–7 and 10–11 (total of 16 crop rows / plot), we deployed two shingles per row (approximately 5 and 10 m from the western edge of each plot; total of four shingles per plot). These crop rows were chosen to reduce the risk of spillovers of slugs from neighboring plots. We removed any crop residue and laid shingles flat on the ground between crop rows.^25^, ^30^ In cover crop plots, we clipped away foliage to allow shingles to rest on soil and then placed the foliage on top of the shingle to best match the moisture and temperature conditions of the matted cover crop. We began counting slug populations under traps immediately following crop planting until corn growth stage V6 (stages when plants are most vulnerable to damage)^31^ and then began again from R5 through harvest (when slugs begin to become adults). To increase efficiency, we checked slugs under shingles in corn and soybean on the same days. In doing so, soybean growth stages varied. For early season, sampling ranged from planting‐V2/3 and late season ranged from R2/3‐harvest. To sample the coolest parts of the day, we conducted assessments as early in the morning as possible (6:00–8:00 AM). For each evaluation, we counted all slugs underneath each shingle and the few that occurred on top of shingles.

### 2.3 Predator community and predation

To evaluate effects of timing of cover‐crop termination on abundance and diversity of ground‐dwelling predators, we deployed two pitfall traps near the center of each plot (5 and 10 m from the western edge of each plot) during 2022 and 2023 in corn and soybeans. Trap location was chosen to reduce the risk of spillovers of predators from neighboring plots. Traps consisted of 7.6 × 7.6 cm (height × diameter) plastic cups about one quarter full of a 50:50 mixture of propylene glycol and water. We covered each trap with plastic plates perched on nails (7.6 cm tall) to prevent rain and debris from falling into traps.^25^, ^32^ We installed traps so they were flush to the ground to allow easy access to soil‐surface dwelling arthropods. We left traps in the field for 48 h and then collected them and returned the contents to the lab to sort arthropods. In 2022, we deployed pitfall traps monthly, starting shortly after crop planting (28 May 2022) and extending until crop maturity (growth stage R5‐6; final trapping date for corn: 1 July 2022; final trapping date for soybeans: 18 August 2022). Final sampling dates in 2022 differed between corn and soybean due to sampling errors. In 2023, we conducted this assay monthly in both corn and soybean, starting post‐plant (corn stage V3; 26 June 2023) until crop maturity (growth stage R5‐6; 15 September 2023). On 15 Sept 2023, many traps were disrupted by vertebrate pests (presumably raccoons, *Procyon lotor*), so we excluded those samples from analyses.

To assess effects of timing of cover‐crop termination on predation by generalist invertebrate predators, we conducted sentinel prey assays with waxworms (*Galleria mellonella*; Timberline Fisheries, Marion, IL) in corn and soybean.^25^, ^33^ For simplicity, both corn and soybeans were sampled on the same dates and times a day before we collected pitfall traps. We pinned waxworms through their terminal segment into balls of modelling clay (Plastilina Modeling Clay, color: cream from Sargent Art, Inc.), which we laid on the soil surface. We deployed six waxworms per plot, with each waxworm in a vertebrate exclusion cage made from hardware cloth (10 mm mesh) and capped with a plastic lid that allowed for free passage of predatory invertebrate species while excluding mice and birds. We deployed three waxworms per row at ~8:00 in two rows of each plot (between rows 6 and 7 and 11 and 12 of corn or soybean, approximately 4, 8, and 12 m from the western edge of each plot; total of six cages per plot). We selected location of sentinel prey cages to reduce risk of spillovers of predators from neighboring plots. We checked on caterpillars at ~20:00 and again the next day at ~8:00, recording presence/absence and noting any predators (e.g., wolf spider, carabid beetle, ant, etc.) that were present. Missing waxworms were not replaced with living ones. For simplicity, both corn and soybeans were sampled on the same dates and times (Supporting Information, Table S2).

## 3 STATISTICAL ANALYSIS

We conducted all statistical analyses in R (version 4.3.1). To analyze effects of cover‐crop termination timing, we used generalized linear mixed‐effect models (GLMM; ‘lme4’ package; Beta‐distribution models: ‘glmmTMB’ package)^34^, ^35^ for the following types of data and fitted distributions: cover‐crop biomass (Gaussian), yield (Gaussian), damage incidence (grouped binomial), damage score (grouped binomial), damage type (binomial), slug counts (negative binomial), and sentinel prey (beta). In a few cases for sentinel prey, assays within experimental units resulted in complete predation, in which proportion predated was truncated to 0.99 to permit fitting of beta‐distributed models. Cover‐crop biomass and yield were assessed with a fixed effect term of treatment*year to capture the main effects and their interaction. For both data types, the random effect term was block.

To determine the significance of fixed main effects and interactions, we used the ‘anova’ function to obtain log‐likelihood ratio tests and the Type‐II Wald Χ^2^ test statistic. The ANOVA output allowed comparisons of null models to fully fitted models, adding one effect at a time. For linear models, we ran a post‐hoc analyses with the ‘emmeans’ package.^36^ Full models were used during the post‐hoc analyses, although the terms used for each respective data type depended on the Wald Χ^2^ output.

We fit treatment, timing (i.e., growth stage), and their interaction as fixed effects to assess damage incidence, damage score, damage type, and sentinel prey. Temporal autocorrelation among damage assessments was accounted for using a plot identifier, which was nested within block and year. For damage rating, we nested the random effects term within year. For damage incidence, damage type and sentinel prey, the random effect term was plot nested within block within year. For slugs, we structured the fixed effect term to capture the main effects and interactions between treatment and year (treatment*year), and the random effect term was block. Due to an imbalance in sampling effort, slug data were averaged across year and treatment and split between spring (planting‐V6) and fall (R3‐crop maturity). This allowed us to keep the treatment*growth stage interaction in the model, while maintaining year and block as sources of autocorrelation. To reduce overcomplication of our models, we chose to exclude crop‐sequence.

To analyze differences in arthropod communities by crop, sampling date, and treatment sampled with pitfall traps, we used permutational multivariate analysis of variance (PERMANOVA; ‘vegan’ package).^37^ We used Bray–Curtis dissimilarity to analyze count data and set permutations to 999. We aggregated predators into four main groups: Araneomorph, Carabidae, Ensifera, and Formicidae. These taxa represent the four most common predator groups recorded in 2022 and 2023. Furthermore, aggregations were made because we were not interested in individual species or genus outcomes. Rather, we were interested in the effects of unique feeding guilds. Indeed, this aggregation is coarse, but it accurately represents the most prominent feeding guilds identified in our sampling. We also analyzed Lycosidae populations within the Araneomorph suborder.

We assessed relationships between pest and predator populations *via* their abundance with a generalized linear model (GLM, Negative binomial distribution; ‘MASS’ package).^38^ The values used in this model were averages of populations by treatments across the 2 years of predator sampling. The response variable was slug abundance and the fixed effect was predator abundance. Random effects were not included in this model, as autocorrelation was not a concern.

To analyze the differences in populations of Carabidae, Araneomorphae, and Lycosidae between crops, we ran GLM (Negative binomial distribution) models with crop as the fixed effect term.

## 4 RESULTS

### 4.1 Cover‐crop biomass

In corn, within each year we found differences in cover‐crop biomass among all treatments except in 2023 (Supporting Information, Fig. S2). For 2021 and 2022, planting green had the greatest biomass, followed by the late‐terminated, and then the early‐terminated treatment. In 2023, planting green and the late‐terminated treatments did not differ in biomass, but were both greater than the early‐terminated treatment. It is important to note; however, the cover‐crop planting dates differed by year, which led to variations in biomass amounts (Supporting Information, Table S1).

In soybean, in 2022 all three cover‐crop treatments were significantly different from each other. Planting green had the greatest biomass, followed by late‐terminated, and then the early‐terminated treatment. In 2023, like our results with corn, the early‐terminated treatment had significantly lower biomass than both late‐terminated and planting‐green treatments, which were similar to each other (Supporting Information, Fig. S3).

### 4.2 Herbivores and their damage

#### 4.2.1 Corn

##### 4.2.1.1 Slugs

Slug populations in spring differed by year; 2021 had the greatest slug populations, 2022 the lowest, while 2023 was intermediate (*Χ* ^2^ = 211, df = 2, *P* < 0.001; Supporting Information, Fig. S4). During 2021, the planting‐green treatment had significantly greater slug populations than the no‐cover‐crop treatment while the early‐ and late‐terminated treatments were intermediate. In both 2022 and 2023, there were no recorded differences among treatments.

**Figure 1:**
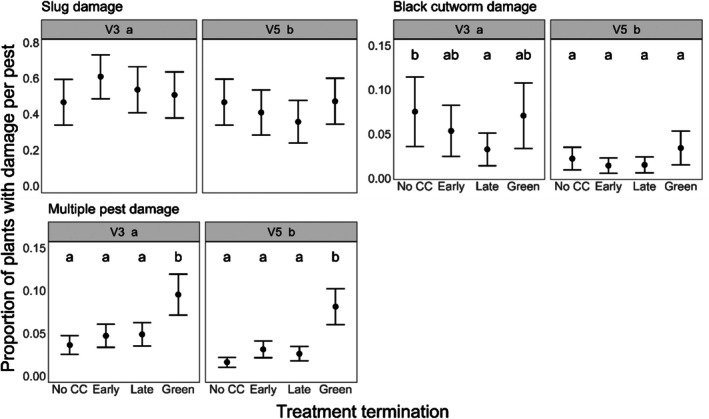
Predicted mean incidence of three types of herbivore damage to corn plants that resulted in statistically significant differences in either treatment, growth stage, or the interaction between treatment and growth stage (see text for statistical details; error bars are response‐scale predictions +/− standard errors from model output). **Slug damage:** Incidence of slug damage (proportion of plants with damage) by growth stage and treatment. There are no differences at the treatment level. **Black cutworm damage:** Incidence of black cutworm damage by growth stage and treatment. **Multiple pest damage:** Incidence of plants with multiple types of pest damage by growth stage and treatment. Different lowercase letters indicate statistical differences among treatments and/or between growth stages (Early: 14–28 DPP; Late: 9–14 DPP; Green: 0–3 DAP) based on post‐hoc comparisons.

For the proportion of plants with slug damage, there was a significant main effect of growth stage (*Χ* ^2^ = 51.09, df = 2, *P* < 0.0001) and the interaction of treatment and growth stage (*Χ* ^2^ = 40.1, df = 3, *P* < 0.0001; Fig. 1(A)). Growth stage V3 had significantly higher incidence of slug damage than V5. Although there were no differences at the treatment level, during both V3 and V5 growth stages, the no‐cover‐crop and planting‐green treatments had similar slug damage values. Additionally, during growth stage V3, there was a higher tendency of damage than V5.

##### 4.2.1.2 Other pest species

For the proportion of plants with black cutworm damage, there was a marginal main effect of treatment (*Χ* ^2^ = 7.64, df = 3, *P* = 0.054) and a significant main effect of growth stage (*Χ* ^2^ = 63.04, df = 1, *P* < 0.0001; Fig. 1(B)). While marginal, for growth stage V3, the no‐cover‐crop treatment had greater damage than the late‐terminated treatment, while the early‐terminated and planting‐green treatments were intermediate. Growth stage V3 had significantly higher incidence of cutworm damage than V5. For armyworm and stink bug damage, there were no differences between growth stages or among treatments. When we explored plants that had multiple types of pest damage, we detected a significant effect of treatment (*Χ* ^2^ = 22.1, df = 3, *P* < 0.0001) and growth stage (*Χ* ^2^ = 13.32, df = 1, *P* = 0.0002; Fig. 1(C)), but not for their interaction (*P* > 0.05). Overall, during both V3 and V5 growth stages, the planting‐green treatment had significantly higher levels of multiple‐pest damage than the three other treatments. Additionally, growth stage V3 had higher incidence of multiple pest damage than V5.

##### 4.2.1.3 Pest damage

**Figure 2:**
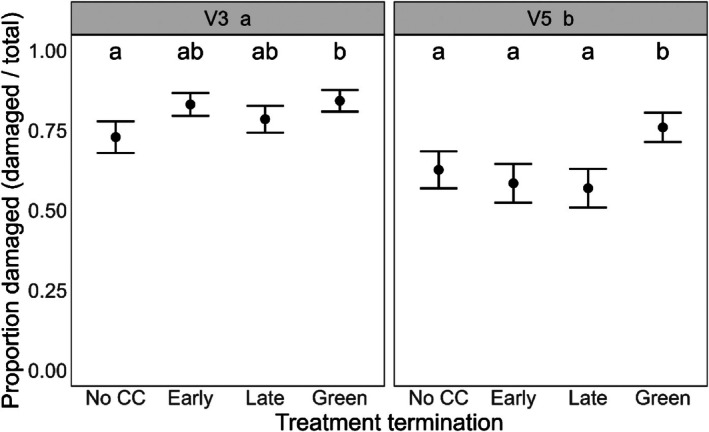
Proportion of pest‐damaged plants per treatment by growth stage (error bars are response‐scale predictions +/− standard errors from model output). Different lowercase letters indicate statistical differences among treatments and/or between growth stages (Early: 14–28 DPP; Late: 9–14 DPP; Green: 0–3 DAP) based on post‐hoc comparisons.

When comparing the proportion of damaged plants across treatments, there was a significant interaction of treatment and growth stage (*Χ* ^2^ = 29.7, df = 3, *P* < 0.0001; Fig. 2), but also the main effect of treatment (*Χ* ^2^ = 11.6, df = 3, *P* = 0.009) and growth stage (*Χ* ^2^ = 189.4, df = 1, *P* < 0.0001). Overall, growth stage V3 had a higher proportion of damaged plants than V5. Within growth stage V3, the planting‐green treatment had a significantly higher proportion of plants damaged than the no‐cover‐crop treatment, while early‐terminated and late‐terminated treatments were intermediate (Fig. 2(A)). Within growth stage V5, the planting‐green treatment had a higher proportion of damaged plants than the three other treatments (Fig. 2(B)).

**Figure 3:**
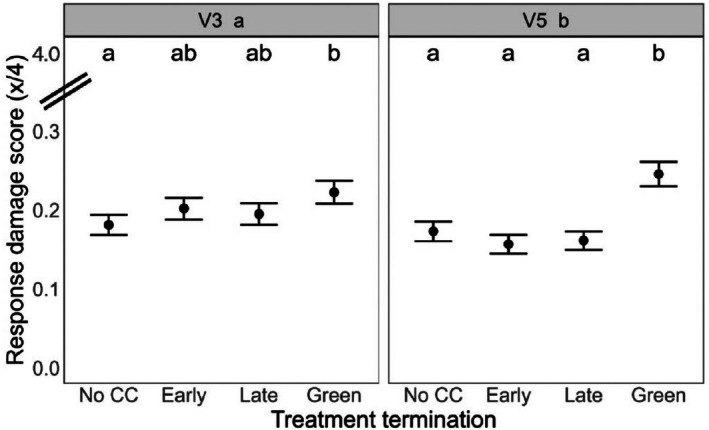
Average damage score for years 2021–2023 in corn (error bars are response‐scale predictions +/− standard errors from model output). Different lowercase letters indicate statistical differences among treatments and/or between growth stages (Early: 14–28 DPP; Late: 9–14 DPP; Green: 0–3 DAP) based on post‐hoc comparisons.

For average damage rating, there were significant main effects of treatment (*Χ* ^2^ = 23.2, df = 3, *P* < 0.0001), growth stage (*Χ* ^2^ = 11.9, df = 1, *P* < 0.0001), and the interaction of treatment and growth stage (*Χ* ^2^ = 31.5, df = 3, *P* < 0.0001; Fig. 3). Growth stage V3 had a greater average damage rating than V5. Within V3 growth stage, corn plants in the planting‐green treatment had the highest average damage rating, which was significantly higher than the damage rating for corn in the no‐cover‐crop treatment, while the early‐ and late‐terminated treatments were intermediate (Fig. 3(A)). For growth stage V5, the damage rating of corn plants in the planting‐green treatment was the highest and significantly higher than each of the other three treatments (Fig. 3(B)).

#### 4.2.2 Soybean

##### 4.2.2.1 Slugs

In soybean, where we did not assess damage to the crop, slug populations in spring differed by year (populations in 2023 were greater than 2022; *Χ* ^2^ = 6, df = 1, *P* = 0.01; Supporting Information, Fig. S5), but not by treatment. For either year, variability at the treatment level was low.

### 4.3 Predator community and predation

#### 4.3.1 Sentinel prey

**Figure 4:**
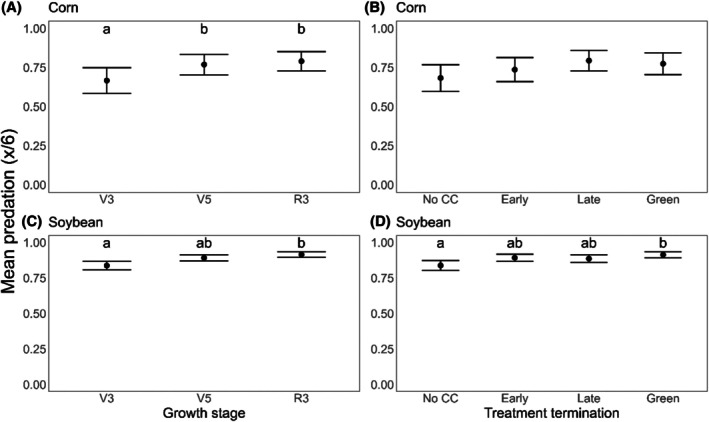
Predicted mean rates of predation on sentinel caterpillars by (A) growth stage in Corn, (B) treatment in Corn, (C) growth stage in Soybean, and (D) treatment in Soybean (error bars are response‐scale predictions +/− standard errors from model output). Different lowercase letters indicate statistical differences among treatments and/or between growth stages (Early: 14–28 DPP; Late: 9–14 DPP; Green: 0–3 DAP) based on post‐hoc comparisons.

In corn, predation rates were significantly affected by growth stage (*Χ* ^2^ = 10.5, df = 2, *P* = 0.005; Fig. 4(A)). Growth stage V3 had fewer prey attacked than V5 and R3, but attack rates during V5 and R3 were similar. At the treatment level, amounts of predation were similar across treatments (Fig. 4(B)). Although not significant, there appears to be a trend of more predation with later terminated cover crops.

In soybean, predation was significantly affected by the interaction of treatment and growth stage (*Χ* ^2^ = 24.5, df = 8, *P* = 0.001; Fig. 4(C), (D)). During growth stage V3, fewer prey were attacked than during growth stage R3 but attack rates during V5 and R3 were similar (Fig. 4(C)). During V3, the no‐cover‐crop treatment had lower rates of predation than the planting‐green treatment, while early‐terminated and late‐terminated treatments were intermediate (Fig. 4(D)).

#### 4.3.2 Pitfall traps

**Figure 5:**
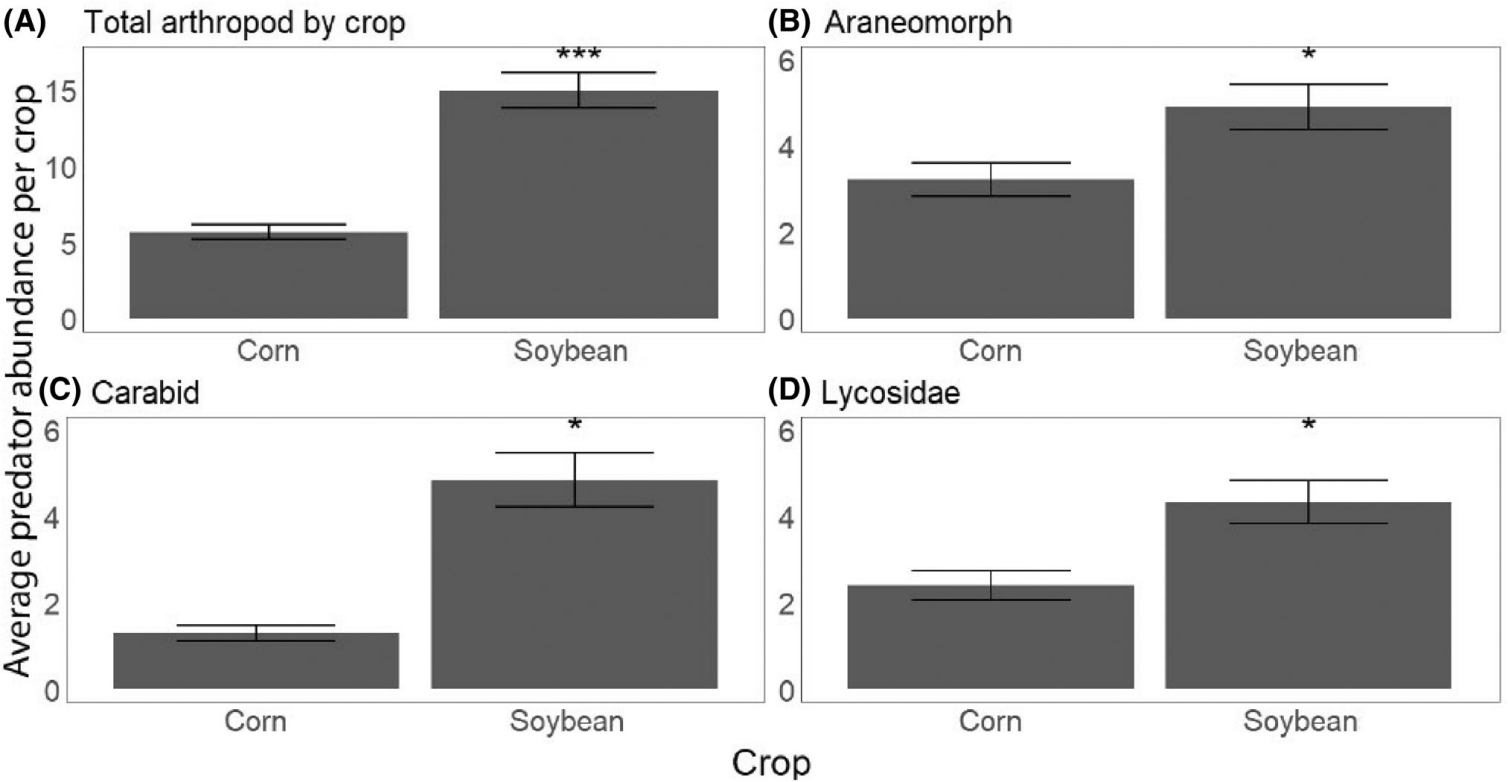
Mean abundance of arthropod predators by crop (error bars are response‐scale predictions +/− standard errors from model output): (A) all predatory arthropods, (B) Araneomorph, (C) Carabid, (D) Lycosidae populations. Asterisks denote significance. (* = *P ≤* 0.05; ** = *P ≤* 0.01; *** = *P ≤* 0.001).

Both crop (*F*_*1,79*_ < 0.001) and sampling date (*F*_*2,79*_ < 0.001) influenced arthropod populations in pitfall traps. Soybean harbored greater populations of arthropods than corn (Fig. 5(A)). When evaluating araneomorphs, carabids, and Lycosidae abundance by crop, all three groups had greater abundance in soybean than corn (Fig. 5(B)–(D)). At the plot level, we recorded similar predatory arthropod abundance in 2022 (Mean ± SE: 10.5 ± 0.7, n = 80) and 2023 (10.1 ± 1.30, n = 80). Araneomorph (40%) and carabid (30%) populations accounted for most of the predators collected. Lycosidae accounted for 83% of the araneomorph populations while carabid was dominated by the genera *Chlaenius* (56%), *Pterostichus* (26%), and *Poecilus* (11%).

##### 4.3.2.1 Corn

**Figure 6:**
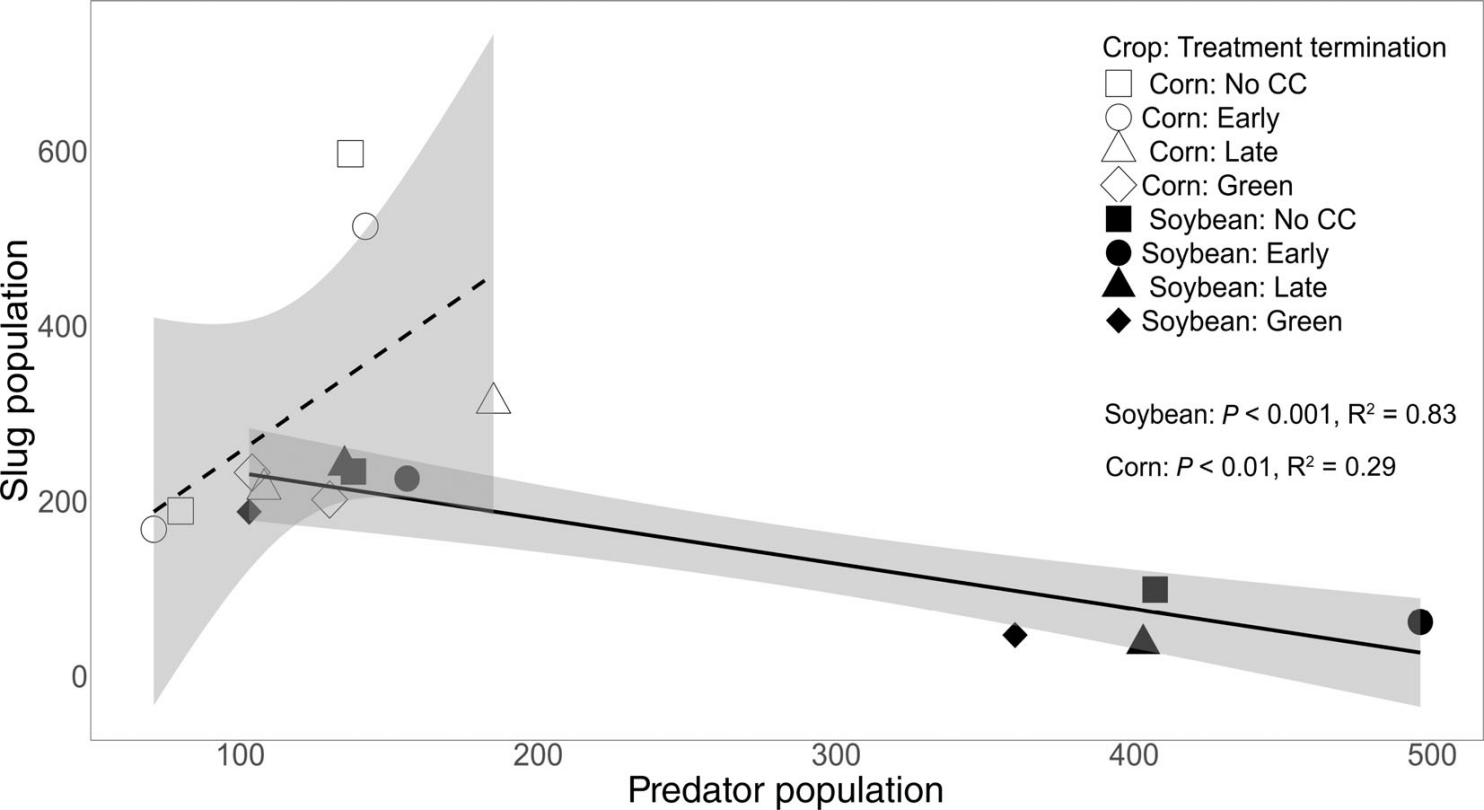
Relationships between slug and arthropod predator populations in corn and soybean. The results presented are from a generalized linear model for non‐parametric data. Each point represents a treatment mean from either 2022 or 2023. Corn is represented by the dashed line with hollow points that represent each treatment; soybean is represented by the solid line and filled points that represent each treatment (Early: 14–28 DPP; Late: 9–14 DPP; Green: 0–3 DAP). The shaded area around the lines represent the 95% confidence limit.

In corn, both year and sampling date affected abundance of predatory arthropod populations (*F*_*1,79*_ < 0.001), whereas treatment had no effect. In 2022 and 2023, we recorded no differences in araneomorph or carabid populations by sampling date. When comparing total abundance of araneomorph and carabids from 2022 and 2023, we found lower (*P* ≤ 0.001) araneomorph populations in 2023 (Supporting Information, Table S3). Additionally, we recorded a significant positive relationship between abundance of slugs and predator (*R* ^2^ = 0.29, *P* < 0.01; Fig. 6).

##### 4.3.2.2 Soybean

In soybean, year and sampling date affected distributions of predatory arthropod populations (*F*_*3,99*_ < 0.001), whereas treatment had no effect. In 2022, we recorded no differences in abundance of araneomorphs by sampling date. Carabids were more abundant during the last sampling date, 18 August 2022, than the two earlier dates (28 May 2022 and 1 July 2022; Supporting Information, Table S4). In 2023, araneomorph abundance was similar among sampling dates. Carabids were more abundant during the last sampling date (28 July 2023) than the first sampling date (26 June 2023; Supporting Information, Table S5). Carabid and araneomorph abundance varied between 2022 (May 28 and July 1) and 2023 (June 26 and July 28; Supporting Information, Table S6). Furthermore, we recorded a negative relationship between abundance of slugs and predators (*R* ^2^ = 0.83, *P* < 0.001 Fig. 6).

### 4.4 Yield

In corn, yield was affected by the interaction between treatment and year (*Χ* ^2^ = 18, df = 6, *P* = 0.006; Supporting Information, Fig. S6), but also the main effects of treatment (*Χ* ^2^ = 13.7, df = 3, *P* = 0.003) and year (*Χ* ^2^ = 8.5, df = 2, *P* = 0.014). In years 2021 and 2022 we recorded no differences in yield among treatments, although there was more variation in the no‐cover‐crop treatment than the other three. In 2023, the no‐cover‐crop treatment had lower yields than the other three treatments, and although the planting‐green treatment was statistically similar to the early‐ and late‐terminated treatments, it had a greater yield.

For soybean, yield was not affected by treatment but was influenced by year (*Χ* ^2^ = 5.9, df = 1, *P* = 0.015; Supporting Information, Fig. S7). The 2022 season had lower overall yields than 2023. Additionally, while there were no treatment differences in 2023, the three cover‐crop treatments appeared to outperform the no‐cover‐crop treatment.

## 5 DISCUSSION

The aim of this study was to evaluate effects of the timing of cover‐crop termination on abundance of pests and predators within corn and soybean systems. Using three termination timings, we succeeded in establishing a gradient of cover‐crop biomass, but our results characterizing the influence of that biomass on pest and predator populations did not fully meet our expectations. First, with greater amounts of cover‐crop biomass, we found little to no increase in damage by invertebrate pests. Second, in corn, we found some variation in herbivore populations and their damage, but damage did not exceed economic thresholds in any of the treatments. Third, for the proportion of plants damaged or the average damage score in each treatment, the planting‐green treatment received among the highest levels of damage during the V3 and V5 growth stages (Figs 2 and 3), but this damage did not relate to yield (Figs S6 and S7). For predation, we found no differences among treatments in corn, whereas in soybeans the planting‐green treatment hosted greater amounts of predation than no‐cover‐crop plots. Moreover, soybeans hosted higher predator populations than corn, leading to lower slug populations in soybeans across all treatments. In summary, our results are nuanced but mostly support our hypotheses that planting green can reduce pest abundance and their damage. It is important to note we did not quantify pest damage in soybean. As a result, our conclusions are appropriate for corn but based on our experience growing soybean in the area,^25^, ^26^ it seems likely that our results are also relevant for soybeans, particularly given the abundance of natural enemies that we measured in soybean plots. Lastly, because our plots did not have borders on either side, we recognize that there may have been some movement of invertebrates between plots, but we expect that our sampling mostly reflected communities of each plot. In larger‐scale, commercial farm fields, such spillover would be less relevant.

### 5.1 Herbivores and their damage

One of the concerns about planting green is that greater amounts of biomass can maintain soil moisture and limit soil temperatures, creating good habitat for pests including caterpillars and slugs, among others.^15^, ^24^, ^39^ During the 3 years of this experiment, our study sites did not experience significant deviations from typical abiotic conditions. Across treatments, we found similar low levels of damage from black cutworms and stink bugs, and we found no damage from true armyworms. For black cutworm, the planting‐green treatment had levels of damage similar to the no‐cover‐crop treatment, suggesting that the planting‐green treatments did not set the subsequent corn crop up for greater damage from caterpillars.^15^ These results are notable because black cutworms and armyworms are often cited by farmers and crop consultants as threats to field corn following cereal rye cover crops and pinpointed as reasons to avoid using grass cover crops. When corn plants suffered attacks from multiple pest species, however, the planting‐green treatment had the highest levels of damage (Fig. 1(C)). Nevertheless, damage remained low and, based on economic thresholds (pest‐specific either formal or informal thresholds) for the individual pest species (black cutworm [Economic Threshold: 5% of seedlings at V3], true armyworm [ET: 25% of plants damaged], stink bugs [ET: 10% of plants infested], and slugs [informal ET: >2 slug per shingle])^31^ did not appear to be economically problematic as less than 10% of corn plants were damaged.

For slugs, which in the mid‐Atlantic region are perennially among the most challenging invertebrate pests faced by no‐till farmers,^3^ we found no differences among treatments in slug damage that plants received (Fig. 1(A)). Growers often assume that cover crops make slug populations worse, but our previous research has shown otherwise.^16^, ^25^ Moreover, it feels natural to suspect that higher levels of cover‐crop biomass could help increase slug populations, resulting in greater damage from slugs, but as found previously,^16^, ^40^ we did not find a consistent relationship between cover‐crop biomass and slug populations or their damage. Rather than timing of cover‐crop termination influencing slug populations in corn, it appears that annual variation in abiotic conditions had the greatest effect on slugs. Spring temperatures and rainfall are drivers of slug populations, which tend to be active in mid‐to‐late spring and early summer when corn and soybean plants are young and most vulnerable to slug feeding.^3^, ^21^, ^22^ In our experiment, however, slug populations were either not large enough to negatively influence early season crop plants or abiotic conditions did give their populations an advantage. It is becoming evident that damage from slugs and their influence on crop productivity by the end of the season depends heavily on abiotic conditions throughout the season, which can influence how well crops recover from slug damage.^41^

### 5.2 Predation and predator communities

In soybean, predation of sentinel caterpillars generally increased with cover‐crop biomass (Fig. 4), consistent with our hypothesis. Indeed, predation during our sampling period at soybean growth stage R3 was nearly 100% in the planting‐green treatment, whereas the no‐cover‐crop treatment predation rate was roughly 80%. These results confirm that increasing complexity in agricultural fields can increase predator activity.^4^, ^6^ Moreover, these results indicate that cover‐crop systems can build robust predator communities and that preventative‐pest‐management strategies may not be necessary in high‐cover biomass systems, where they could be disruptive.^29^, ^42^ Rather, when combined with previous research, the current results suggest that regular scouting and use of economic thresholds as part of an IPM program can protect predator communities so that they can contribute to pest control.^25^, ^26^, ^43^

In addition to greater prey consumption with higher cover‐crop biomass, predation also increased throughout the season. In soybean plots, predation at growth stage V3 (~16–22 June) was significantly lower than at growth stages V5 (~24 June‐15 July) and R3 (~24 August – 15 September). This increase in predation as the season progressed was likely driven by a broad predator community that can build with time and can include spiders, ants, adults and/or larvae of some beetle species (e.g., Carabidae, Lampyridae, Staphylinidae).^26^ Carabid beetles are often key predators in no‐till systems and adults of key species, including *Pterostichus melanarius*, are active mid‐summer into autumn and can account for a majority of predation in our region^44^; however, carabid populations appeared equal for many of our sampling dates (Supporting Information, Table S3).

Regardless of treatment, our soybean plots harbored greater predator populations than corn plots and, as a result, predators in soybeans appear to have driven down slug populations (Fig. 6). Soybean fields can have key characteristics that help build natural‐enemy communities, particularly their high plant populations (typically >345 000 plants/ha) that canopy early in the summer within and between rows to create humid microclimates, and higher population canopy and increased humidity earlier with benefits for beneficial arthropods.^45^ In contrast, corn fields tend to be less hospitable for natural enemies because they have a lower number of plants per acre (around 74 000 plants/ha) and fewer leaves per plant that do not strongly interdigitate with adjacent plants to create a dense canopy of leaves. While there are likely to be multiple factors at play, we hypothesize that the corn canopy tends to allow more sun to reach the soil surface, leading to lower biodiversity.^6^ Notably, the more robust predator communities harbored in soybeans can control key soybean pests.^29^, ^46^, ^47^ Moreover, the results presented here suggest that high cover‐crop biomass systems can also foster predator communities that can limit slug populations and can complement bottom‐up control that can be active in these systems.^40^

The contrasting relationships between predators and slugs in soybeans and corn is intriguing and suggests factors that could be harnessed to drive natural‐enemy communities in various types of agricultural fields. Perhaps crop fields planted with higher densities of plants that canopy well might provide similar hospitable environments for natural enemies like those produced by soybean.^45^ For example, there is much current interest in short‐stature corn, which is less susceptible to wind damage.^48^ If adopted, short‐stature corn would be planted at higher populations^49^ that would seem to canopy earlier and block sunlight from reaching soil, factors that could create more hospitable conditions below the canopy for natural enemies. High density plantings of annual crops may generally provide benefits to natural‐enemy abundance, but for crop production to gain from such communities growers would probably need to embrace integrated pest management (IPM) and avoid preventative insecticide use. In our experiment, we purposely avoided insecticides because previous research has revealed that using IPM can help grow natural‐enemy communities that can contribute significantly to pest control of invertebrate pests.^26^, ^50^

### 5.3 Yield

Importantly, we found that the high‐biomass treatments did not reduce crop yield. Rather, yields were either neutral or increased with more cover‐crop biomass (Supporting Information, Figs S6 and S7). Similar yield across the four treatments in both corn and soybean is consistent with the findings of previous research from the same region.^24^ This trend corroborates our hypothesis that treatments with high cover‐crop biomass can produce competitive yields, a finding that may help increase adoption of planting‐green practices.

When combined, no‐till and planting green have potential to improve functioning of annual agronomic ecosystems. Higher levels of cover‐crop biomass can significantly suppress weed populations, including those of problematic, herbicide‐resistant species.^51^ Moreover, the results presented here indicate that planting green, in the absence of insecticide use, can build natural‐enemy communities that can be an effective first line of defense against invertebrate pests.^25^, ^26^ To maximize the pest‐management potential of such conservation‐based systems, however, growers would need to adopt IPM to avoid preventative insecticide applications, including seed‐applied insecticides, to allow natural‐enemy communities to grow. This combination of no‐till, cover crops, and IPM can lead to a high diversity‐IPM system in which crop plants are protected by natural enemies, which in some cases are more effective than control provided by insecticides.^25^, ^26^ By practicing high diversity‐IPM, growers can reduce damage from invertebrate herbivores to their crops, increase natural‐enemy populations, and maintain, if not improve, economic and environmental sustainability of annual row‐crop systems.^25^, ^27^

## 6 CONCLUSIONS

Despite concerns in agronomic systems of increasing pest populations and reducing yield by planting green,^3^, ^15^ our results suggest that planting green: (i) may not increase pest damage or damage incidence, (ii) can promote predation, and (iii) may not limit yield in either corn or soybean. In the mid‐Atlantic region, studies have demonstrated the benefits that come from planting green and practicing IPM.^1^, ^12^, ^24^, ^40^ These benefits include, but are not limited to, reducing potential for soil erosion,^9^, ^17^ improving soil quality,^9^ decreasing erosion and nutrient leaching into watersheds,^1^, ^18^, ^19^ and decreasing weed and invertebrate pest pressures, which is supported by our findings and previous studies.^2^, ^3^, ^4^, ^5^, ^6^, ^7^, ^8^, ^20^, ^24^, ^40^ It is also worth noting that while we terminated cereal rye using herbicides, cover crops can also be killed by mowing or crimping. Previous research indicated that methods of termination can differentially influence pest and natural‐enemy communities^39^; therefore, such a line of inquiry could not be pursued for planting green, but the research could be valuable given concerns about herbicides in the environment.^52^ Our results suggest that these practices could be adopted in the Mid‐Atlantic region and beyond by row‐crop growers looking to increase ecosystem services in their crop fields while decreasing reliance on insecticides.

## CONFLICT OF INTEREST

The authors declare no conflicts of interest.

## Supporting information


**Table S1.** Cover‐crop and cash‐crop planting and termination dates and seeding rates.
**Table S1a**. Soil type information for the three field sites and the associated crops and year. All soil data retrieved from the Web Soil Survey (Soil Survey Staff, n.d.).
**Table S2**. Sentinel prey assessment dates with corn and soybean growth stages.
**Table S3**. Araneomorph and carabid averages by sampling date from corn pitfalls. This table comprises both 2022 corn sampling dates (28 May and 1 July) and both 2023 sampling dates (26 June and 28 July). Araneomorphs had greater populations in 2022 than 2023.
**Table S4**. Araneomorph and carabid averages by 2022 sampling dates of pitfalls from soybean plots. Carabid populations increase among the three sampling dates from 2022.
**Table S5**. Araneomorph and carabid averages by 2023 sampling dates of pitfalls from soybean plots. Carabid populations increase between the two sampling dates from 2023.
**Table S6**. Araneomorph and carabid averages by sampling date of pitfalls from soybean plots. This table comprises two of the 2022 sampling dates (28 May and 1 July) and both 2023 sampling dates (26 June and 28 July). Araneomorphs had greater abundance in 2022 than 2023. Carabids had the highest abundance during 28 July 2023.
**Fig. S1**. Schematic diagram of the design showing treatments, blocks, and buffer areas. (Early: 14–28 DPP; Late: 9–14 DPP; Green: 0–3 DAP).
**Fig. S2**. Cover‐crop biomass from corn plots split by treatment and year. In 2021 and 2022, all cover‐crop treatments differed. In 2023, early‐terminated was lower than the late‐terminated and planting‐green treatments, but late‐terminated and planting‐green treatments did not differ. Letter change denotes significance. (Early: 14–28 DPP; Late: 9–14 DPP; Green: 0–3 DAP).
**Fig. S3**. Cover‐crop biomass from soybean plots split by treatment and year. In 2022, all cover‐crop treatments differed. In 2023, early‐terminated was lower than the late‐terminated and planting‐green treatments, but late‐terminated and planting‐green treatments did not differ. Letter change denotes significance. (Early: 14–28 DPP; Late: 9–14 DPP; Green: 0–3 DAP).
**Fig. S4**. Slugs populations by year and treatment from the spring season in corn plots. There was a significant main effect of year (*Χ* ^2^ = 211, df = 2, *P* < 0.001). The 2021 season had greater slug populations than the 2022 season but did not differ from 2023. During 2021, the planting‐green treatment had greater slug populations than the no‐cover‐crop check treatment but did not differ from either the early‐terminated or the late‐terminated treatments. Letter change denotes significance. (Early: 14–28 DPP; Late: 9–14 DPP; Green: 0–3 DAP).
**Fig. S5**. Slugs populations by year and treatment from the spring season in soybean plots. There was a significant main effect of year (*Χ* ^2^ = 6, df = 1, *P* = 0.01) for slug populations sampled during spring. Slug populations were greater in 2023 than 2022 Letter change denotes significance. (Early: 14–28 DPP; Late: 9–14 DPP; Green: 0–3 DAP).
**Fig. S6**. Yield in bushels/acre from corn plots. there was a significant main effect of treatment (*Χ* ^2^ = 13.7, df = 3, *P* = 0.003), the interaction between treatment and year (*Χ* ^2^ = 18, df = 6, *P* = 0.006), and year (*Χ* ^2^ = 8.5, df = 2, *P* = 0.014). In years 2021 and 2022 I recorded no differences in yield among treatments. In 2023, the no‐cover‐crop check was lower than the other three treatments. Letter change denotes significance. (Early: 14–28 DPP; Late: 9–14 DPP; Green: 0–3 DAP).
**Fig. S7**. Yield in bushels / acre from soybean plots. There was a significant main effect of year (*Χ* ^2^ = 5.9, df = 1, *P* = 0.015). The 2022 season had lower overall yields than 2023. Letter change denotes significance. (Early: 14–28 DPP; Late: 9–14 DPP; Green: 0–3 DAP).

## Data Availability

The data that support the findings of this study are openly available in PSA_Pennsylvania at https://github.com/Jared-Adam/PSA_Pennsylvania.
