# Supplementary material for: Managing pests by increasing predators through late termination of cover crops
Source: Pest Manag Sci. 2026 May 20;82(9):8477–87. doi: 10.1002/ps.70898 (PMC13453194; doi:10.1002/ps.70898)
Supplement: Supplementary file 1 [file PS-82-8477-s001.docx]

**Supporting information:**

**Tables:**

| **Table S1**. Cover-crop and cash-crop planting and termination dates and seeding rates. | | | | | | | | | | | | | | | | | | |
| --- | --- | --- | --- | --- | --- | --- | --- | --- | --- | --- | --- | --- | --- | --- | --- | --- | --- | --- |
|  | |  | |  | | **Treatment termination** | | | | |  | |  | |  | | |  |
| **Crop year** | **Corn field cover crop planting date** | | **Soybean field cover crop planting date** | | **Cereal rye seeding rate (kg/ha)** | | **No Cover Crop** | **Early** | **Late** | **Planting green** | | **Corn planting date** | | **Corn seeding rate (seeds/ha)** | | **Soybean planting date** | **Soybean seeding rate (seeds/ha)** | |
| 2021 | 8 Sept. 2020 | | - | | 67.5 | | - | 27 April | 12 May | 24 May | | 21 May | | 81,250 | | - | - | |
| 2022 | 10 Nov. 2021 | | 21 Oct. 2021 | | 67.5 | | - | 29 April | 17 May | 1 June | | 31 May | | 81,250 | | 31 May | 450,000 | |
| 2023 | 26 Oct. 2022 | | 10 Nov. 2022 | | 67.5 | | - | 5 May | 12 May | 23 May | | 23 May | | 81,250 | | 23 May | 450,000 | |

| **Table S1a**. Soil type information for the three field sites and the associated crops and year. All soil data retrieved from the Web Soil Survey (Soil Survey Staff, n.d.). | | | | | | | | | |
| --- | --- | --- | --- | --- | --- | --- | --- | --- | --- |
|  |  |  |  |  |  |  |  |  |  |
| **Field number** | **Crop** | **Year** | **Crop** | **Year** | **Soil Type** | **Slope** | **%** | **Slope** | **%** |
| 1 | Corn | 2021 | Soybean | 2022 | Hagerstown silt loam | 0 to 3 | 66.7 | 3 to 8 | 33.3 |
| 2 | Corn | 2022 | Soybean | 2023 | Murrill channery silt loam | 0 to 3 | 100 | - | - |
| 3 | Corn | 2023 | - | - | Murrill channery silt loam | 0 to 3 | 100 | - | - |

| **Table S2**. Sentinel prey assessment dates with corn and soybean growth stages. | | |
| --- | --- | --- |
| **Date** | **Corn Growth Stage** | **Soybean Growth Stage** |
| 6/16/2021 | V3 | V2-3 |
| 6/24/2021 | V5 | V4 |
| 8/24/2021 | R3 | R4 |
| 6/22/2022 | V3 | V2-3 |
| 7/12/2022 | V5 | V4 |
| 9/1/2022 | R3 | R4 |
| 6/20/2023 | V3 | V2-3 |
| 7/15/2023 | V5 | V4 |
| 9/15/2023 | R3 | R4 |

| **Table S3.** Araneomorph and carabid averages by sampling date from corn pitfalls. This table comprises both 2022 corn sampling dates (28 May and 1 July) and both 2023 sampling dates (26 June and 28 July). Araneomorphs had greater populations in 2022 than 2023.  **CLD; Compact letter display:** Letter change denotes significance.  **N = 20** | | | | |  |
| --- | --- | --- | --- | --- | --- |
| **Sampling date** | **Araneomorph mean ± SE** | **Araneomorph CLD** | **Carabid mean ± SE** | **Carabid CLD** | |
| 28 May 2022 | 4.6 ± 0.6 | a | 1.4 ± 0.5 | a | |
| 1 July 2022 | 6.7 ± 0.7 | a | 0.6 ± 0.2 | a | |
| 26 June 2023 | 0.7 ± 0.2 | b | 1.15 ± 0.3 | a | |
| 28 July 2023 | 0.9 ± 0.3 | b | 2.00 ± 0.4 | a | |

| **Table S4.** Araneomorph and carabid averages by 2022 sampling dates of pitfalls from soybean plots. Carabid populations increase among the three sampling dates from 2022.  **CLD; Compact letter display:** Letter change denotes significance.  **N = 20** | | | | |
| --- | --- | --- | --- | --- |
| **Sampling date** | **Araneomorph mean ± SE** | **Araneomorph CLD** | **Carabid mean ± SE** | **Carabid CLD** |
| 28 May 2022 | 6.5 ± 1.3 | a | 3.7 ± 0.7 | a |
| 1 July 2022 | 8.75 ± 0.9 | a | 4.6 ± 0.6 | a |
| 18 August 2022 | 11.90 ± 4.9 | a | 17.5 ± 2.8 | b |

| **Table S5.** Araneomorph and carabid averages by 2023 sampling dates of pitfalls from soybean plots. Carabid populations increase between the two sampling dates from 2023.  **CLD; Compact letter display:** Letter change denotes significance.  **N = 20** | | | | |
| --- | --- | --- | --- | --- |
| **Sampling date** | **Araneomorph mean ± SE** | **Araneomorph CLD** | **Carabid mean ± SE** | **Carabid CLD** |
| 26 June 2023 | 1.8 ± 0.3 | a | 2.5 ± 1.5 | a |
| 28 July 2023 | 2.55 ± 0.5 | a | 8.1 ± 1.8 | b |

| **Table S6.** Araneomorph and carabid averages by sampling date of pitfalls from soybean plots. This table comprises two of the 2022 sampling dates (28 May and 1 July) and both 2023 sampling dates (26 June and 28 July). Araneomorphs had greater abundance in 2022 than 2023. Carabids had the highest abundance during 28 July 2023.  **CLD; Compact letter display:** Letter change denotes significance.  **N = 20** | | | | |
| --- | --- | --- | --- | --- |
| **Sampling date** | **Araneomorph mean ± SE** | **Araneomorph CLD** | **Carabid mean ± SE** | **Carabid CLD** |
| 28 May 2022 | 6.5 ± 1.3 | a | 3.7 ± 0.7 | a |
| 1 July 2022 | 8.8 ± 0.9 | a | 4.6 ± 0.6 | ab |
| 26 June 2023 | 1.8 ± 0.3 | b | 2.5 ± 1.5 | a |
| 28 July 2023 | 2.6 ± 0.5 | b | 8.1 ± 1.8 | b |

**Figures:**

**Figure S1.** Schematic diagram of the design showing treatments, blocks, and buffer areas. (Early: 14-28 DPP; Late: 9-14 DPP; Green: 0-3 DAP)

**Figure S2**. Cover-crop biomass from corn plots split by treatment and year. In 2021 and 2022, all cover-crop treatments differed. In 2023, early-terminated was lower than the late-terminated and planting-green treatments, but late-terminated and planting-green treatments did not differ. Letter change denotes significance. (Early: 14-28 DPP; Late: 9-14 DPP; Green: 0-3 DAP)

**Figure S3**. Cover-crop biomass from soybean plots split by treatment and year. In 2022, all cover-crop treatments differed. In 2023, early-terminated was lower than the late-terminated and planting-green treatments, but late-terminated and planting-green treatments did not differ. Letter change denotes significance. (Early: 14-28 DPP; Late: 9-14 DPP; Green: 0-3 DAP)

**Figure S4.** Slugs populations by year and treatment from the spring season in corn plots. There was a significant main effect of year (*Χ^2^*=211, df=2, *P-value*<0.001). The 2021 season had greater slug populations than the 2022 season but did not differ from 2023. During 2021, the planting-green treatment had greater slug populations than the no-cover-crop check treatment but did not differ from either the early-terminated or the late-terminated treatments. Letter change denotes significance. (Early: 14-28 DPP; Late: 9-14 DPP; Green: 0-3 DAP)

**Figure S5.** Slugs populations by year and treatment from the spring season in soybean plots. There was a significant main effect of year (*Χ^2^*=6, df=1, *P-value*=0.01) for slug populations sampled during spring. Slug populations were greater in 2023 than 2022 Letter change denotes significance. (Early: 14-28 DPP; Late: 9-14 DPP; Green: 0-3 DAP)

**Figure S6.** Yield in bushels / acre from corn plots. there was a significant main effect of treatment (*Χ^2^*=13.7, df=3, *P-value*=0.003), the interaction between treatment and year (*Χ^2^*=18, df=6, *P-value*=0.006), and year (*Χ^2^*=8.5, df=2, *P-value*=0.014). In years 2021 and 2022 I recorded no differences in yield among treatments. In 2023, the no-cover-crop check was lower than the other three treatments. Letter change denotes significance. (Early: 14-28 DPP; Late: 9-14 DPP; Green: 0-3 DAP)

**Figure S7.** Yield in bushels / acre from soybean plots. There was a significant main effect of year (*Χ^2^*=5.9, df=1, *P-value*=0.015). The 2022 season had lower overall yields than 2023. Letter change denotes significance. (Early: 14-28 DPP; Late: 9-14 DPP; Green: 0-3 DAP)
